# Supplementary material for: Impact of Tyrosine Kinase Inhibitors on the Expression Pattern of Epigenetic Regulators
Source: Cancers (Basel). 2025 Apr 10;17(8):1282. doi: 10.3390/cancers17081282 (PMC12025482; doi:10.3390/cancers17081282)
Supplement: Supplementary file 1 [file cancers-17-01282-s001.zip › S3.pptx]

## Slide 1
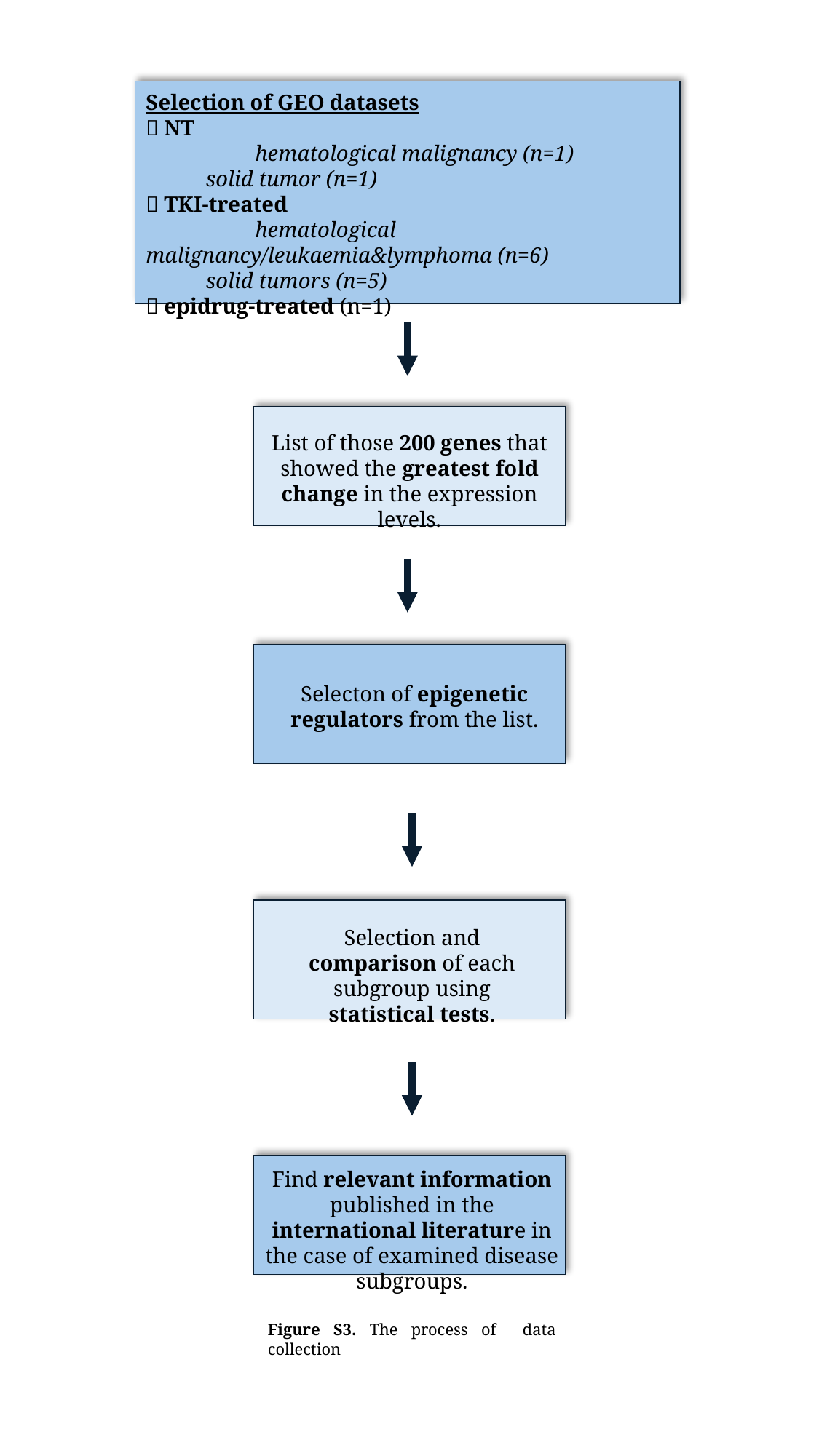

Selection of GEO datasets
 NT
	hematological malignancy (n=1)
 solid tumor (n=1)
 TKI-treated
	hematological malignancy/leukaemia&lymphoma (n=6)
 solid tumors (n=5)
 epidrug-treated (n=1)
List of those 200 genes that showed the greatest fold change in the expression levels.
Selecton of epigenetic regulators from the list.
Selection and comparison of each subgroup using statistical tests.
Find relevant information published in the international literature in the case of examined disease subgroups.
Figure S3. The process of data collection
