# Supplementary material for: Impact of Tyrosine Kinase Inhibitors on the Expression Pattern of Epigenetic Regulators
Source: Cancers (Basel). 2025 Apr 10;17(8):1282. doi: 10.3390/cancers17081282 (PMC12025482; doi:10.3390/cancers17081282)
Supplement: Supplementary file 1 [file cancers-17-01282-s001.zip › S1.pptx]

## Slide 1
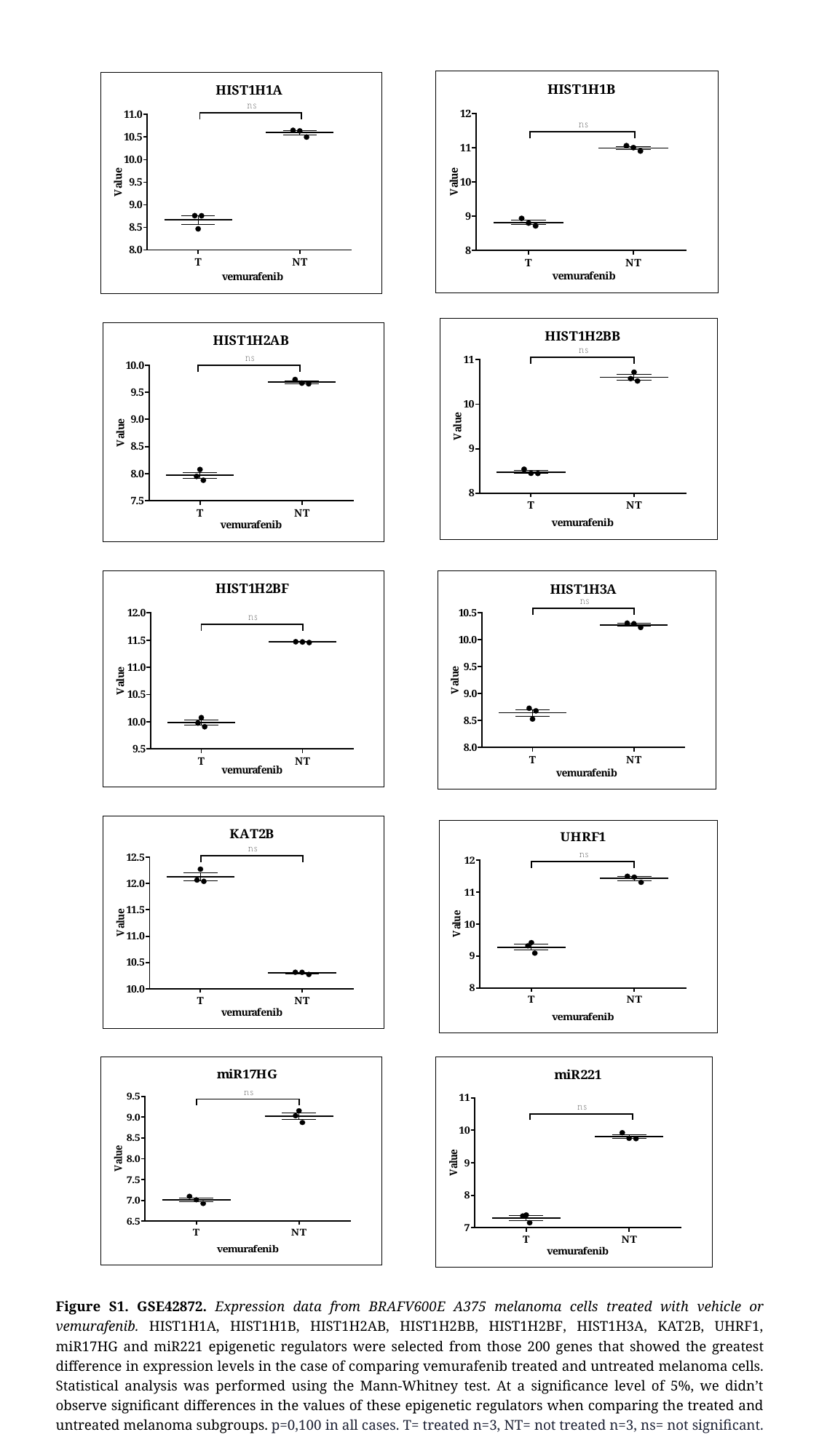

Figure S1. GSE42872. Expression data from BRAFV600E A375 melanoma cells treated with vehicle or vemurafenib. HIST1H1A, HIST1H1B, HIST1H2AB, HIST1H2BB, HIST1H2BF, HIST1H3A, KAT2B, UHRF1, miR17HG and miR221 epigenetic regulators were selected from those 200 genes that showed the greatest difference in expression levels in the case of comparing vemurafenib treated and untreated melanoma cells. Statistical analysis was performed using the Mann-Whitney test. At a significance level of 5%, we didn’t observe significant differences in the values ​​of these epigenetic regulators when comparing the treated and untreated melanoma subgroups. p=0,100 in all cases. T= treated n=3, NT= not treated n=3, ns= not significant.
