# Supplementary material for: Impact of Tyrosine Kinase Inhibitors on the Expression Pattern of Epigenetic Regulators
Source: Cancers (Basel). 2025 Apr 10;17(8):1282. doi: 10.3390/cancers17081282 (PMC12025482; doi:10.3390/cancers17081282)
Supplement: Supplementary file 1 [file cancers-17-01282-s001.zip › S2.pptx]

## Slide 1
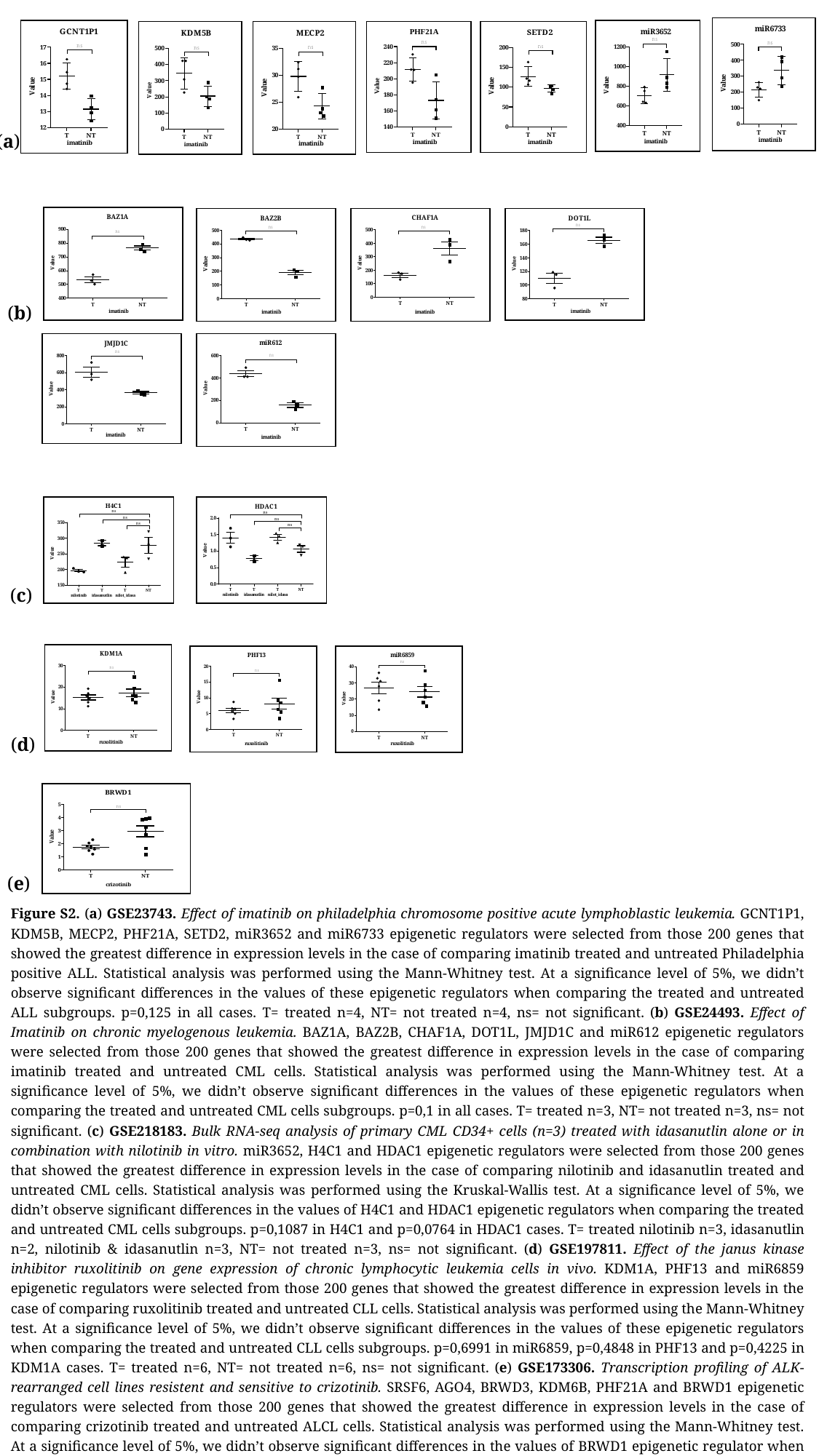

(a)
(b)
(c)
(d)
(e)
Figure S2. (a) GSE23743. Effect of imatinib on philadelphia chromosome positive acute lymphoblastic leukemia. GCNT1P1, KDM5B, MECP2, PHF21A, SETD2, miR3652 and miR6733 epigenetic regulators were selected from those 200 genes that showed the greatest difference in expression levels in the case of comparing imatinib treated and untreated Philadelphia positive ALL. Statistical analysis was performed using the Mann-Whitney test. At a significance level of 5%, we didn’t observe significant differences in the values ​​of these epigenetic regulators when comparing the treated and untreated ALL subgroups. p=0,125 in all cases. T= treated n=4, NT= not treated n=4, ns= not significant. (b) GSE24493. Effect of Imatinib on chronic myelogenous leukemia. BAZ1A, BAZ2B, CHAF1A, DOT1L, JMJD1C and miR612 epigenetic regulators were selected from those 200 genes that showed the greatest difference in expression levels in the case of comparing imatinib treated and untreated CML cells. Statistical analysis was performed using the Mann-Whitney test. At a significance level of 5%, we didn’t observe significant differences in the values ​​of these epigenetic regulators when comparing the treated and untreated CML cells subgroups. p=0,1 in all cases. T= treated n=3, NT= not treated n=3, ns= not significant. (c) GSE218183. Bulk RNA-seq analysis of primary CML CD34+ cells (n=3) treated with idasanutlin alone or in combination with nilotinib in vitro. miR3652, H4C1 and HDAC1 epigenetic regulators were selected from those 200 genes that showed the greatest difference in expression levels in the case of comparing nilotinib and idasanutlin treated and untreated CML cells. Statistical analysis was performed using the Kruskal-Wallis test. At a significance level of 5%, we didn’t observe significant differences in the values ​​of H4C1 and HDAC1 epigenetic regulators when comparing the treated and untreated CML cells subgroups. p=0,1087 in H4C1 and p=0,0764 in HDAC1 cases. T= treated nilotinib n=3, idasanutlin n=2, nilotinib & idasanutlin n=3, NT= not treated n=3, ns= not significant. (d) GSE197811. Effect of the janus kinase inhibitor ruxolitinib on gene expression of chronic lymphocytic leukemia cells in vivo. KDM1A, PHF13 and miR6859 epigenetic regulators were selected from those 200 genes that showed the greatest difference in expression levels in the case of comparing ruxolitinib treated and untreated CLL cells. Statistical analysis was performed using the Mann-Whitney test. At a significance level of 5%, we didn’t observe significant differences in the values ​​of these epigenetic regulators when comparing the treated and untreated CLL cells subgroups. p=0,6991 in miR6859, p=0,4848 in PHF13 and p=0,4225 in KDM1A cases. T= treated n=6, NT= not treated n=6, ns= not significant. (e) GSE173306. Transcription profiling of ALK-rearranged cell lines resistent and sensitive to crizotinib. SRSF6, AGO4, BRWD3, KDM6B, PHF21A and BRWD1 epigenetic regulators were selected from those 200 genes that showed the greatest difference in expression levels in the case of comparing crizotinib treated and untreated ALCL cells. Statistical analysis was performed using the Mann-Whitney test. At a significance level of 5%, we didn’t observe significant differences in the values ​​of BRWD1 epigenetic regulator when comparing the treated and untreated ALCL cells subgroups. p=0,0842. T= treated n=7, NT= not treated n=7, T= treated n=7, NT= not treated n=7, ns= not significant
